# Supplementary material for: Meta-Analysis and Gene Set Enrichment Relative to ER Status Reveal Elevated Activity of MYC and E2F in the “Basal” Breast Cancer Subgroup
Source: PLoS One. 2009 Mar 9;4(3):e4710. doi: 10.1371/journal.pone.0004710 (PMC2650420; doi:10.1371/journal.pone.0004710)
Supplement: Dataset S1 — GSEA results from first screen. The file contains a zipped archive of the HTML results of the second GSEA screen. Reports for enriched or depleted datasets may be accessed from the “index.html” link within the my_analysis.GseaPreranked.1219975950625.rpt.zip ZIP archive. Enrichment plots are available for each of the 50 most enriched and depleted gene sets. (13.32 MB ZIP) [file pone.0004710.s001.zip › my_analysis.GseaPreranked.1219975950625.rpt.zip!//TARTE_PLASMA_BLASTIC.html]

Details for gene set TARTE\_PLASMA\_BLASTIC[GSEA]

|  || Dataset | zSco.ERneg.to.ERpos\_collapsed |
| Phenotype | NoPhenotypeAvailable |
| Upregulated in class | na\_pos |
| GeneSet | TARTE\_PLASMA\_BLASTIC |
| Enrichment Score (ES) | 0.52335304 |
| Normalized Enrichment Score (NES) | 2.4824555 |
| Nominal p-value | 0.0 |
| FDR q-value | 0.0 |
| FWER p-Value | 0.0 |
Table: GSEA Results Summary

  

Fig 1: Enrichment plot: TARTE\_PLASMA\_BLASTIC      
 Profile of the Running ES Score & Positions of GeneSet Members on the Rank Ordered List

  

| PROBE | GENE SYMBOL | GENE\_TITLE | RANK IN GENE LIST | RANK METRIC SCORE | RUNNING ES | CORE ENRICHMENT || 1 | UQCRH |  |  | 5 | 7.406 | 0.0094 | Yes |
| 2 | CDC20 |  |  | 9 | 7.151 | 0.0187 | Yes |
| 3 | TTK |  |  | 11 | 7.059 | 0.0280 | Yes |
| 4 | MSN |  |  | 20 | 6.766 | 0.0363 | Yes |
| 5 | FOXM1 |  |  | 21 | 6.760 | 0.0453 | Yes |
| 6 | CCNE1 |  |  | 24 | 6.716 | 0.0540 | Yes |
| 7 | LBR |  |  | 30 | 6.601 | 0.0624 | Yes |
| 8 | CENPA |  |  | 51 | 6.315 | 0.0693 | Yes |
| 9 | CCNA2 |  |  | 52 | 6.297 | 0.0776 | Yes |
| 10 | NASP |  |  | 59 | 6.201 | 0.0854 | Yes |
| 11 | STIL |  |  | 60 | 6.200 | 0.0936 | Yes |
| 12 | LDHB |  |  | 82 | 6.005 | 0.0999 | Yes |
| 13 | TRIP13 |  |  | 96 | 5.890 | 0.1068 | Yes |
| 14 | MKI67 |  |  | 98 | 5.871 | 0.1145 | Yes |
| 15 | CTPS |  |  | 123 | 5.706 | 0.1202 | Yes |
| 16 | KIF2C |  |  | 124 | 5.703 | 0.1277 | Yes |
| 17 | UCK2 |  |  | 132 | 5.685 | 0.1347 | Yes |
| 18 | SNRPG |  |  | 142 | 5.628 | 0.1415 | Yes |
| 19 | KIFC1 |  |  | 156 | 5.525 | 0.1478 | Yes |
| 20 | YBX1 |  |  | 157 | 5.523 | 0.1552 | Yes |
| 21 | GGH |  |  | 161 | 5.504 | 0.1622 | Yes |
| 22 | RFC3 |  |  | 164 | 5.493 | 0.1694 | Yes |
| 23 | PSME4 |  |  | 175 | 5.460 | 0.1758 | Yes |
| 24 | MTHFD2 |  |  | 205 | 5.307 | 0.1807 | Yes |
| 25 | ORC1L |  |  | 212 | 5.280 | 0.1872 | Yes |
| 26 | KIF14 |  |  | 221 | 5.215 | 0.1935 | Yes |
| 27 | BIRC5 |  |  | 229 | 5.191 | 0.1998 | Yes |
| 28 | SLC7A5 |  |  | 235 | 5.169 | 0.2063 | Yes |
| 29 | CKS2 |  |  | 239 | 5.145 | 0.2129 | Yes |
| 30 | ATP1B3 |  |  | 249 | 5.095 | 0.2190 | Yes |
| 31 | TFRC |  |  | 259 | 5.071 | 0.2250 | Yes |
| 32 | EZH2 |  |  | 312 | 4.888 | 0.2275 | Yes |
| 33 | CDC25B |  |  | 340 | 4.826 | 0.2318 | Yes |
| 34 | RFC4 |  |  | 370 | 4.741 | 0.2359 | Yes |
| 35 | C10ORF7 |  |  | 375 | 4.736 | 0.2418 | Yes |
| 36 | NFIL3 |  |  | 379 | 4.733 | 0.2479 | Yes |
| 37 | GART |  |  | 415 | 4.639 | 0.2514 | Yes |
| 38 | CKS1B |  |  | 416 | 4.638 | 0.2575 | Yes |
| 39 | GZMB |  |  | 466 | 4.470 | 0.2597 | Yes |
| 40 | MAD2L1 |  |  | 477 | 4.446 | 0.2648 | Yes |
| 41 | DLG7 |  |  | 521 | 4.306 | 0.2672 | Yes |
| 42 | CDC27 |  |  | 523 | 4.294 | 0.2728 | Yes |
| 43 | PLK4 |  |  | 524 | 4.292 | 0.2785 | Yes |
| 44 | TUBA1 |  |  | 563 | 4.214 | 0.2812 | Yes |
| 45 | APPBP1 |  |  | 565 | 4.213 | 0.2867 | Yes |
| 46 | CCT6A |  |  | 566 | 4.210 | 0.2923 | Yes |
| 47 | PSMB2 |  |  | 569 | 4.204 | 0.2977 | Yes |
| 48 | XRCC6 |  |  | 581 | 4.186 | 0.3024 | Yes |
| 49 | ATP13A3 |  |  | 587 | 4.179 | 0.3076 | Yes |
| 50 | TARS |  |  | 591 | 4.170 | 0.3129 | Yes |
| 51 | ASNS |  |  | 618 | 4.099 | 0.3163 | Yes |
| 52 | CYCS |  |  | 624 | 4.086 | 0.3213 | Yes |
| 53 | SLC16A1 |  |  | 638 | 4.055 | 0.3257 | Yes |
| 54 | MCM3 |  |  | 656 | 4.019 | 0.3297 | Yes |
| 55 | GPI |  |  | 663 | 4.015 | 0.3346 | Yes |
| 56 | UCHL3 |  |  | 667 | 4.000 | 0.3397 | Yes |
| 57 | LIMS1 |  |  | 709 | 3.913 | 0.3417 | Yes |
| 58 | KIF11 |  |  | 713 | 3.909 | 0.3467 | Yes |
| 59 | COX7B |  |  | 771 | 3.804 | 0.3473 | Yes |
| 60 | TCP1 |  |  | 779 | 3.799 | 0.3518 | Yes |
| 61 | TMPO |  |  | 792 | 3.788 | 0.3559 | Yes |
| 62 | CENPE |  |  | 808 | 3.766 | 0.3598 | Yes |
| 63 | WARS |  |  | 810 | 3.765 | 0.3647 | Yes |
| 64 | TYMS |  |  | 829 | 3.738 | 0.3683 | Yes |
| 65 | CCT7 |  |  | 832 | 3.737 | 0.3731 | Yes |
| 66 | RANBP5 |  |  | 838 | 3.729 | 0.3776 | Yes |
| 67 | ABCE1 |  |  | 882 | 3.649 | 0.3792 | Yes |
| 68 | KIAA0179 |  |  | 885 | 3.648 | 0.3838 | Yes |
| 69 | GINS1 |  |  | 902 | 3.632 | 0.3874 | Yes |
| 70 | FAS |  |  | 922 | 3.609 | 0.3908 | Yes |
| 71 | FDPS |  |  | 949 | 3.555 | 0.3935 | Yes |
| 72 | CHAF1A |  |  | 971 | 3.525 | 0.3965 | Yes |
| 73 | MRPL3 |  |  | 980 | 3.510 | 0.4006 | Yes |
| 74 | DDX39 |  |  | 989 | 3.498 | 0.4046 | Yes |
| 75 | UBE2C |  |  | 994 | 3.489 | 0.4089 | Yes |
| 76 | BOP1 /// LOC653119 |  |  | 1001 | 3.482 | 0.4131 | Yes |
| 77 | KIF23 |  |  | 1030 | 3.442 | 0.4155 | Yes |
| 78 | MRPL19 |  |  | 1061 | 3.401 | 0.4177 | Yes |
| 79 | PSMD14 |  |  | 1069 | 3.392 | 0.4216 | Yes |
| 80 | NDUFA9 |  |  | 1095 | 3.366 | 0.4242 | Yes |
| 81 | SOD2 |  |  | 1111 | 3.347 | 0.4275 | Yes |
| 82 | CCT4 |  |  | 1148 | 3.309 | 0.4291 | Yes |
| 83 | PFKP |  |  | 1165 | 3.281 | 0.4322 | Yes |
| 84 | YARS |  |  | 1177 | 3.262 | 0.4357 | Yes |
| 85 | HSPE1 |  |  | 1183 | 3.257 | 0.4396 | Yes |
| 86 | PRKDC |  |  | 1234 | 3.204 | 0.4400 | Yes |
| 87 | NP |  |  | 1241 | 3.199 | 0.4438 | Yes |
| 88 | SFRS3 |  |  | 1242 | 3.199 | 0.4481 | Yes |
| 89 | CDC2 |  |  | 1259 | 3.182 | 0.4511 | Yes |
| 90 | GALNT3 |  |  | 1265 | 3.174 | 0.4549 | Yes |
| 91 | SFRS10 |  |  | 1320 | 3.118 | 0.4549 | Yes |
| 92 | TMSB10 |  |  | 1333 | 3.100 | 0.4581 | Yes |
| 93 | PCNA |  |  | 1346 | 3.087 | 0.4612 | Yes |
| 94 | S100A10 |  |  | 1435 | 3.014 | 0.4585 | Yes |
| 95 | RAB27A |  |  | 1438 | 3.011 | 0.4623 | Yes |
| 96 | JARID1A |  |  | 1501 | 2.941 | 0.4614 | Yes |
| 97 | TROAP |  |  | 1562 | 2.891 | 0.4607 | Yes |
| 98 | BYSL |  |  | 1577 | 2.872 | 0.4634 | Yes |
| 99 | GNL2 |  |  | 1589 | 2.862 | 0.4663 | Yes |
| 100 | PRPS2 |  |  | 1592 | 2.859 | 0.4700 | Yes |
| 101 | HMMR |  |  | 1608 | 2.843 | 0.4726 | Yes |
| 102 | CCNB1 |  |  | 1626 | 2.823 | 0.4750 | Yes |
| 103 | NKRF |  |  | 1633 | 2.819 | 0.4783 | Yes |
| 104 | KLHDC3 |  |  | 1639 | 2.812 | 0.4817 | Yes |
| 105 | GLDC |  |  | 1680 | 2.772 | 0.4823 | Yes |
| 106 | PSMA1 |  |  | 1687 | 2.766 | 0.4855 | Yes |
| 107 | NEK2 |  |  | 1703 | 2.758 | 0.4880 | Yes |
| 108 | SUMO1 |  |  | 1736 | 2.736 | 0.4891 | Yes |
| 109 | PWP1 |  |  | 1799 | 2.692 | 0.4880 | Yes |
| 110 | CDKN3 |  |  | 1827 | 2.674 | 0.4894 | Yes |
| 111 | RECQL |  |  | 1860 | 2.639 | 0.4905 | Yes |
| 112 | FLNA |  |  | 1895 | 2.609 | 0.4913 | Yes |
| 113 | HUWE1 |  |  | 1899 | 2.607 | 0.4945 | Yes |
| 114 | SNRPC |  |  | 1939 | 2.581 | 0.4950 | Yes |
| 115 | HPRT1 |  |  | 1947 | 2.573 | 0.4978 | Yes |
| 116 | CSE1L |  |  | 1990 | 2.537 | 0.4980 | Yes |
| 117 | RRM2 |  |  | 2047 | 2.485 | 0.4970 | Yes |
| 118 | VDAC1 |  |  | 2049 | 2.484 | 0.5002 | Yes |
| 119 | EBP |  |  | 2055 | 2.478 | 0.5031 | Yes |
| 120 | CD58 |  |  | 2106 | 2.446 | 0.5025 | Yes |
| 121 | SGK |  |  | 2146 | 2.419 | 0.5027 | Yes |
| 122 | PPIF |  |  | 2174 | 2.403 | 0.5038 | Yes |
| 123 | MPHOSPH6 |  |  | 2211 | 2.382 | 0.5042 | Yes |
| 124 | TXN |  |  | 2214 | 2.379 | 0.5072 | Yes |
| 125 | GCLM |  |  | 2267 | 2.339 | 0.5063 | Yes |
| 126 | SSR1 |  |  | 2351 | 2.285 | 0.5030 | Yes |
| 127 | RPA1 |  |  | 2380 | 2.270 | 0.5038 | Yes |
| 128 | CCT5 |  |  | 2398 | 2.262 | 0.5055 | Yes |
| 129 | PAICS |  |  | 2407 | 2.255 | 0.5079 | Yes |
| 130 | EXOSC2 |  |  | 2431 | 2.235 | 0.5091 | Yes |
| 131 | SNRPD3 |  |  | 2432 | 2.233 | 0.5121 | Yes |
| 132 | GLIPR1 |  |  | 2439 | 2.229 | 0.5146 | Yes |
| 133 | EBNA1BP2 |  |  | 2478 | 2.211 | 0.5146 | Yes |
| 134 | SMARCA4 |  |  | 2485 | 2.206 | 0.5170 | Yes |
| 135 | IGHG1 |  |  | 2497 | 2.200 | 0.5191 | Yes |
| 136 | CCND2 |  |  | 2506 | 2.197 | 0.5214 | Yes |
| 137 | CBX5 |  |  | 2621 | 2.133 | 0.5155 | Yes |
| 138 | CORO1A |  |  | 2626 | 2.131 | 0.5180 | Yes |
| 139 | CASP1 |  |  | 2645 | 2.120 | 0.5194 | Yes |
| 140 | SLC19A1 |  |  | 2675 | 2.105 | 0.5200 | Yes |
| 141 | SLA |  |  | 2700 | 2.092 | 0.5209 | Yes |
| 142 | LMNB1 |  |  | 2764 | 2.060 | 0.5188 | Yes |
| 143 | GIT2 |  |  | 2773 | 2.057 | 0.5209 | Yes |
| 144 | AIM1 |  |  | 2807 | 2.037 | 0.5211 | Yes |
| 145 | LGALS1 |  |  | 2838 | 2.020 | 0.5215 | Yes |
| 146 | ATP5J |  |  | 2885 | 1.992 | 0.5206 | Yes |
| 147 | PRKCB1 |  |  | 2915 | 1.980 | 0.5210 | Yes |
| 148 | DHFR /// LOC643509 / |  |  | 2981 | 1.938 | 0.5185 | Yes |
| 149 | IDH2 |  |  | 3005 | 1.929 | 0.5193 | Yes |
| 150 | KYNU |  |  | 3045 | 1.911 | 0.5189 | Yes |
| 151 | ITGB7 |  |  | 3076 | 1.896 | 0.5191 | Yes |
| 152 | PAK2 |  |  | 3098 | 1.887 | 0.5200 | Yes |
| 153 | PSMB8 |  |  | 3139 | 1.871 | 0.5194 | Yes |
| 154 | BAK1 /// BCL2L7P1 |  |  | 3142 | 1.869 | 0.5217 | Yes |
| 155 | CDC6 |  |  | 3185 | 1.847 | 0.5209 | Yes |
| 156 | MTHFD1 |  |  | 3258 | 1.814 | 0.5178 | Yes |
| 157 | CCNF |  |  | 3265 | 1.809 | 0.5197 | Yes |
| 158 | TNFSF7 |  |  | 3357 | 1.768 | 0.5151 | Yes |
| 159 | VBP1 |  |  | 3382 | 1.758 | 0.5156 | Yes |
| 160 | MAPK6 |  |  | 3396 | 1.751 | 0.5169 | Yes |
| 161 | MAPK1 |  |  | 3460 | 1.726 | 0.5143 | Yes |
| 162 | CD180 |  |  | 3469 | 1.723 | 0.5160 | Yes |
| 163 | TOPBP1 |  |  | 3475 | 1.722 | 0.5179 | Yes |
| 164 | TDG |  |  | 3528 | 1.695 | 0.5162 | Yes |
| 165 | PDAP1 |  |  | 3533 | 1.693 | 0.5181 | Yes |
| 166 | NDUFS1 |  |  | 3541 | 1.689 | 0.5198 | Yes |
| 167 | TAX1BP3 |  |  | 3545 | 1.687 | 0.5218 | Yes |
| 168 | IFI30 |  |  | 3555 | 1.684 | 0.5234 | Yes |
| 169 | IL2RB |  |  | 3652 | 1.639 | 0.5182 | No |
| 170 | PSMD4 |  |  | 3664 | 1.636 | 0.5195 | No |
| 171 | EIF3S10 |  |  | 3722 | 1.614 | 0.5172 | No |
| 172 | NNT |  |  | 3734 | 1.610 | 0.5185 | No |
| 173 | TOP2A |  |  | 3735 | 1.610 | 0.5207 | No |
| 174 | HADH2 |  |  | 3873 | 1.565 | 0.5122 | No |
| 175 | HMGB2 |  |  | 3883 | 1.558 | 0.5136 | No |
| 176 | ATP5O |  |  | 3901 | 1.554 | 0.5143 | No |
| 177 | PSMA5 |  |  | 3924 | 1.544 | 0.5147 | No |
| 178 | PTPN1 |  |  | 4004 | 1.511 | 0.5106 | No |
| 179 | DBI |  |  | 4012 | 1.506 | 0.5121 | No |
| 180 | SMC1A |  |  | 4069 | 1.485 | 0.5098 | No |
| 181 | SELL |  |  | 4075 | 1.483 | 0.5113 | No |
| 182 | OAT |  |  | 4103 | 1.470 | 0.5112 | No |
| 183 | UBE2L3 |  |  | 4118 | 1.465 | 0.5121 | No |
| 184 | UFD1L |  |  | 4248 | 1.414 | 0.5040 | No |
| 185 | LILRB4 |  |  | 4498 | 1.320 | 0.4867 | No |
| 186 | TKT |  |  | 4548 | 1.303 | 0.4846 | No |
| 187 | MRPL12 |  |  | 4601 | 1.286 | 0.4823 | No |
| 188 | SNRPB2 |  |  | 4602 | 1.286 | 0.4840 | No |
| 189 | IQGAP1 |  |  | 4609 | 1.283 | 0.4853 | No |
| 190 | PSCDBP |  |  | 4664 | 1.264 | 0.4828 | No |
| 191 | FH |  |  | 4665 | 1.264 | 0.4845 | No |
| 192 | ACOT7 |  |  | 4673 | 1.261 | 0.4856 | No |
| 193 | KIAA0101 |  |  | 4770 | 1.230 | 0.4799 | No |
| 194 | GARS |  |  | 4828 | 1.211 | 0.4771 | No |
| 195 | MYO5A |  |  | 4850 | 1.206 | 0.4771 | No |
| 196 | KIF2A |  |  | 4858 | 1.203 | 0.4782 | No |
| 197 | SNRPF |  |  | 4861 | 1.201 | 0.4796 | No |
| 198 | RRM1 |  |  | 4872 | 1.197 | 0.4804 | No |
| 199 | PAPOLA |  |  | 4942 | 1.175 | 0.4767 | No |
| 200 | PSMC3IP |  |  | 5109 | 1.113 | 0.4654 | No |
| 201 | EED |  |  | 5267 | 1.060 | 0.4547 | No |
| 202 | SLC39A7 |  |  | 5312 | 1.043 | 0.4527 | No |
| 203 | CD52 |  |  | 5341 | 1.031 | 0.4520 | No |
| 204 | GNB1 |  |  | 5358 | 1.024 | 0.4521 | No |
| 205 | LDLR |  |  | 5398 | 1.012 | 0.4504 | No |
| 206 | TCEB1 |  |  | 5401 | 1.010 | 0.4516 | No |
| 207 | NARS |  |  | 5486 | 0.978 | 0.4465 | No |
| 208 | U2AF2 |  |  | 5499 | 0.975 | 0.4468 | No |
| 209 | POLD2 |  |  | 5531 | 0.964 | 0.4457 | No |
| 210 | COPS5 |  |  | 5596 | 0.945 | 0.4421 | No |
| 211 | HPGD |  |  | 5622 | 0.938 | 0.4414 | No |
| 212 | C6ORF32 |  |  | 5676 | 0.922 | 0.4385 | No |
| 213 | EIF5 |  |  | 5824 | 0.870 | 0.4284 | No |
| 214 | PGK1 |  |  | 5836 | 0.865 | 0.4287 | No |
| 215 | ESPL1 |  |  | 5874 | 0.853 | 0.4270 | No |
| 216 | AHCY |  |  | 6002 | 0.812 | 0.4183 | No |
| 217 | BRD8 |  |  | 6010 | 0.808 | 0.4188 | No |
| 218 | CKAP1 |  |  | 6069 | 0.791 | 0.4154 | No |
| 219 | TIMM17A |  |  | 6273 | 0.724 | 0.4008 | No |
| 220 | CPOX |  |  | 6500 | 0.649 | 0.3843 | No |
| 221 | PRIM1 |  |  | 6525 | 0.642 | 0.3833 | No |
| 222 | DNAJC7 |  |  | 6547 | 0.635 | 0.3825 | No |
| 223 | GPIAP1 |  |  | 6591 | 0.624 | 0.3801 | No |
| 224 | BLMH |  |  | 6669 | 0.599 | 0.3749 | No |
| 225 | SDHB |  |  | 6679 | 0.597 | 0.3750 | No |
| 226 | DUT |  |  | 6839 | 0.539 | 0.3635 | No |
| 227 | PSMA2 |  |  | 6855 | 0.533 | 0.3631 | No |
| 228 | ITGB1 |  |  | 7054 | 0.467 | 0.3485 | No |
| 229 | USP14 |  |  | 7091 | 0.452 | 0.3463 | No |
| 230 | ELMO1 |  |  | 7189 | 0.422 | 0.3394 | No |
| 231 | FOXK2 |  |  | 7318 | 0.377 | 0.3301 | No |
| 232 | OSTF1 |  |  | 7320 | 0.377 | 0.3305 | No |
| 233 | ACTA2 |  |  | 7342 | 0.369 | 0.3294 | No |
| 234 | ETF1 |  |  | 7421 | 0.338 | 0.3239 | No |
| 235 | TMEM97 |  |  | 7575 | 0.280 | 0.3125 | No |
| 236 | ATP5F1 |  |  | 7640 | 0.254 | 0.3079 | No |
| 237 | CBX3 /// LOC653972 |  |  | 7691 | 0.234 | 0.3044 | No |
| 238 | PSMA3 |  |  | 7737 | 0.218 | 0.3012 | No |
| 239 | AFF1 |  |  | 7753 | 0.212 | 0.3003 | No |
| 240 | NME1 |  |  | 7807 | 0.187 | 0.2965 | No |
| 241 | RB1 |  |  | 8000 | 0.105 | 0.2819 | No |
| 242 | MRPL23 |  |  | 8086 | 0.076 | 0.2755 | No |
| 243 | CLIC1 |  |  | 8141 | 0.060 | 0.2714 | No |
| 244 | EIF2S1 |  |  | 8146 | 0.058 | 0.2712 | No |
| 245 | NDUFB7 |  |  | 8277 | 0.007 | 0.2612 | No |
| 246 | ANP32A |  |  | 8388 | -0.031 | 0.2528 | No |
| 247 | TRAP1 |  |  | 8457 | -0.058 | 0.2476 | No |
| 248 | SUMO2 |  |  | 8509 | -0.083 | 0.2438 | No |
| 249 | CCT2 |  |  | 8514 | -0.085 | 0.2436 | No |
| 250 | TOP1 |  |  | 8568 | -0.109 | 0.2397 | No |
| 251 | CLEC2B |  |  | 8628 | -0.141 | 0.2353 | No |
| 252 | YWHAH |  |  | 8629 | -0.142 | 0.2355 | No |
| 253 | TUBA3 |  |  | 8675 | -0.160 | 0.2323 | No |
| 254 | FDFT1 |  |  | 8694 | -0.166 | 0.2311 | No |
| 255 | UQCRC2 |  |  | 8708 | -0.175 | 0.2304 | No |
| 256 | KIF22 |  |  | 8725 | -0.180 | 0.2294 | No |
| 257 | HSPA4 |  |  | 8813 | -0.214 | 0.2230 | No |
| 258 | PSME2 |  |  | 8865 | -0.240 | 0.2194 | No |
| 259 | JTV1 |  |  | 9145 | -0.379 | 0.1984 | No |
| 260 | TOP2B |  |  | 9541 | -0.583 | 0.1689 | No |
| 261 | PDLIM1 |  |  | 9543 | -0.584 | 0.1696 | No |
| 262 | UBE2D3 |  |  | 9545 | -0.587 | 0.1703 | No |
| 263 | TUBG1 |  |  | 9551 | -0.592 | 0.1707 | No |
| 264 | HNRPC |  |  | 9598 | -0.616 | 0.1679 | No |
| 265 | IGF1 |  |  | 9639 | -0.637 | 0.1657 | No |
| 266 | BCL3 |  |  | 9660 | -0.650 | 0.1650 | No |
| 267 | EWSR1 |  |  | 9674 | -0.657 | 0.1649 | No |
| 268 | PPP1R2 |  |  | 9753 | -0.695 | 0.1598 | No |
| 269 | GALNT1 |  |  | 9778 | -0.711 | 0.1589 | No |
| 270 | LSP1 /// LOC649377 |  |  | 9817 | -0.735 | 0.1570 | No |
| 271 | GSPT1 |  |  | 9841 | -0.751 | 0.1562 | No |
| 272 | PHB |  |  | 10007 | -0.852 | 0.1447 | No |
| 273 | RNASE6 |  |  | 10023 | -0.858 | 0.1447 | No |
| 274 | MAPKAPK3 |  |  | 10168 | -0.939 | 0.1348 | No |
| 275 | RARS |  |  | 10232 | -0.979 | 0.1313 | No |
| 276 | ARID3A |  |  | 10505 | -1.169 | 0.1120 | No |
| 277 | SCAP |  |  | 10557 | -1.213 | 0.1096 | No |
| 278 | CDKN1A |  |  | 10787 | -1.380 | 0.0939 | No |
| 279 | ENTPD1 |  |  | 10881 | -1.457 | 0.0887 | No |
| 280 | NDUFV2 |  |  | 10912 | -1.487 | 0.0883 | No |
| 281 | BAG1 |  |  | 10930 | -1.508 | 0.0890 | No |
| 282 | CLCN3 |  |  | 11233 | -1.771 | 0.0682 | No |
| 283 | ACVR1 |  |  | 11343 | -1.872 | 0.0623 | No |
| 284 | MYCBP |  |  | 11420 | -1.954 | 0.0590 | No |
| 285 | PPP4C |  |  | 11470 | -2.005 | 0.0579 | No |
| 286 | LOC91137 |  |  | 11486 | -2.018 | 0.0595 | No |
| 287 | CASP6 |  |  | 11499 | -2.032 | 0.0612 | No |
| 288 | ANXA6 |  |  | 11651 | -2.167 | 0.0525 | No |
| 289 | ADD1 |  |  | 11683 | -2.204 | 0.0530 | No |
| 290 | CRADD |  |  | 11698 | -2.218 | 0.0549 | No |
| 291 | ICT1 |  |  | 11750 | -2.268 | 0.0540 | No |
| 292 | HSPA9B |  |  | 11777 | -2.299 | 0.0550 | No |
| 293 | ASNA1 |  |  | 12125 | -2.733 | 0.0320 | No |
| 294 | FASN |  |  | 12283 | -2.945 | 0.0239 | No |
| 295 | ATIC |  |  | 12402 | -3.104 | 0.0189 | No |
| 296 | ARHGDIB |  |  | 12440 | -3.165 | 0.0203 | No |
| 297 | ZNF410 |  |  | 12528 | -3.301 | 0.0180 | No |
| 298 | PSMC5 |  |  | 12531 | -3.303 | 0.0222 | No |
| 299 | IQGAP2 |  |  | 12733 | -3.700 | 0.0116 | No |
| 300 | ABCD3 |  |  | 12788 | -3.840 | 0.0126 | No |
| 301 | COASY |  |  | 12810 | -3.911 | 0.0162 | No |
| 302 | COX6C |  |  | 12819 | -3.930 | 0.0208 | No |
| 303 | ANG /// RNASE4 |  |  | 12867 | -4.053 | 0.0225 | No |
| 304 | MVP |  |  | 13004 | -4.466 | 0.0180 | No |
| 305 | DHCR24 |  |  | 13061 | -4.682 | 0.0199 | No |
Table: GSEA details [plain text format]

  

Fig 2: TARTE\_PLASMA\_BLASTIC: Random ES distribution      
 Gene set null distribution of ES for **TARTE\_PLASMA\_BLASTIC**
